# TMNet: A Two-Branch Multi-Scale Semantic Segmentation Network for Remote Sensing Images

**DOI:** 10.3390/s23135909

**Published:** 2023-06-26

**Authors:** Yupeng Gao, Shengwei Zhang, Dongshi Zuo, Weihong Yan, Xin Pan

**Affiliations:** 1 School of Computer and Information Engineering, Inner Mongolia Agricultural University, Hohhot 010011, China; 2 Inner Mongolia Autonomous Region Key Laboratory of Big Data Research and Application of Agriculture and Animal Husbandry, Hohhot 750306, China; 3 College of Water Conservancy and Civil Engineering, Inner Mongolia Agricultural University, Hohhot 010018, China; 4 Key Laboratory of Water Resources Protection and Utilization of Inner Mongolia Autonomous Region, Hohhot 750306, China; 5 Institute of Grassland Research of CAAS, Hohhot 010013, China

**Keywords:** remote sensing images, global modeling, semantic segmentation, Swin transformer

## Abstract

Pixel-level information of remote sensing images is of great value in many fields. CNN has a strong ability to extract image backbone features, but due to the localization of convolution operation, it is challenging to directly obtain global feature information and contextual semantic interaction, which makes it difficult for a pure CNN model to obtain higher precision results in semantic segmentation of remote sensing images. Inspired by the Swin Transformer with global feature coding capability, we design a two-branch multi-scale semantic segmentation network (TMNet) for remote sensing images. The network adopts the structure of a double encoder and a decoder. The Swin Transformer is used to increase the ability to extract global feature information. A multi-scale feature fusion module (MFM) is designed to merge shallow spatial features from images of different scales into deep features. In addition, the feature enhancement module (FEM) and channel enhancement module (CEM) are proposed and added to the dual encoder to enhance the feature extraction. Experiments were conducted on the WHDLD and Potsdam datasets to verify the excellent performance of TMNet.

## 1. Introduction

In the field of image processing, the research on semantic segmentation of remote sensing images is becoming one of the hot topics nowadays. It classifies each pixel in the image, and the information of the classification results is valuable in many fields, such as land planning [1] and natural disaster assessment [2] and land surveying [3].

Remote sensing images contain a large amount of feature information, and the same type of features present feature diversity and complexity at different times or locations [4]. Compared with car scenes and object images [5,6], the semantic segmentation of remote sensing images is obviously more difficult. Its characteristics of long distance and large scope of shooting make the size of the object features extremely inconsistent, and it is easy to have large intra-class variance and small inter-class variance. Features such as the large range of size variation of the same kind of target, obvious color and texture differences, and difficulty in distinguishing between mixed objects of different kinds mean that remote sensing image segmentation is more difficult compared with other images.

The rapid development of convolutional neural networks (CNNs) has played an important role in promoting research in the field of image processing, but CNN structures are always used for the classification of the whole image rather than for each pixel in the image. The invention of the full convolutional network (FCN) [7] is a milestone for the semantic segmentation of images, replacing the fully connected layer after the CNN with a deconvolutional layer to achieve the upsampling of features, thus allowing the output of per-pixel classification results of the same size for any size of the input image size. U-Net [8] inherits the idea of FCN, deepens the feature upsampling process, and makes its network a ‘U’-shaped symmetric structure to improve the accuracy of image semantic segmentation. SegNet [9] builds an encoder–decoder structure to achieve end-to-end pixel-level image segmentation. However, CNN-based models often result in missing small-scale features during feature extraction using operations such as convolution and pooling [10]. In addition, different classes of objects may have similar size, material, and spectral features in an image, which are difficult to distinguish and cannot be fully recovered in high-resolution detail, especially for class edges [11]. The local nature of the convolution operation hinders the ability of the model to directly interact with contextual information and global modeling [12]. Given the complexity of category features in remote sensing images, more contextual information and global features are needed to support image pixel classification [13]. In order to address the shortcomings of the pure CNN model in extracting global features of remote sensing images performance and enhance its ability to obtain contextual information, we propose a two-branch multiscale semantic segmentation network (TMNet), which differs from classical network structures such as U-Net or Deeplab [14] in that it uses a dual encoder–decoder structure that can balance computational complexity and model parameters while improving model accuracy. Given the great advantage of the CNN model in spatial feature extraction capability, this model uses CNN as the main encoder to extract image backbone features. The Swin Transformer has powerful global feature modeling capability. In this model, the Swin Transformer is used as an auxiliary encoder to compensate for the lack of global feature extraction capability of the CNN to achieve better extraction results. Adding a skip connection between the encoder and decoder increases the contextual information interaction capability and increases the stability of data features. In addition, we proposed a multi-scale fusion module (MFM) to increase the feature fusion at different scales, and we design a feature enhancement module (FEM) and a channel enhancement module (CEM) to enhance the feature extraction capability of the model. The main work of this paper can be summarized by the following three points:

1. A two-branch multi-scale semantic segmentation network (TMNet) for remote sensing images is proposed to improve the model image classification performance by the global feature information encoding capability of the Swin Transformer.
2. The MFM is proposed to merge shallow spatial features of images at different scales into deep features and increase the fusion of multi-scale deep image features of the model to recover more minute details in the classification process.
3. FEM and CEM are added to the main and auxiliary encoders, respectively, to enhance feature extraction. FEM enhances feature information interaction by calculating the relationship between its own feature information and updateable feature storage units; CEM further increases the spatial correlation of global features by establishing an inter-channel correlation to encode the spatial information of Swin Transformer.

The main work of this paper is as follows: Section 1 summarizes the current research status in the field of remote sensing image segmentation and the methodology and contributions of this paper. In Section 2, the related work on the proposed method of this model is presented. Section 3 is a detailed description of this method, and Section 4 is the experimental part, including various parameters, results, and analysis of the experiments. Section 5 is the conclusion of this paper.

## 2. Related Work

### 2.1. Classical Image Semantic Segmentation Network

In recent years, remote sensing image segmentation methods based on the deep learning framework have made great progress. DenseASPP [15] uses dilated convolution with different expansion rates to enhance its global modeling capability. PSPNet [16] uses a pyramid pooling module to acquire features and uses multiple scale pooling operations to realize the fusion of different scale features. DenseU-Net [17] improves the image classification accuracy by deepening the convolution layer and using U-Net architecture to realize the aggregation of small-scale features. BiSeNet [18] is a bilateral segmentation network designed to extract spatial information and generate high-resolution features in small steps, and to obtain sufficient receptive fields using a context path with a fast downsampling strategy. BSNet [19] enhances the global contextual information interaction and boundary information extraction by dynamic gradient convolution and coordinate sensitive attention. HRNet [20] enhances feature fusion by the iterative information exchange of multi-level features and has multiple scales of convolutional combinations to enhance spatial information accuracy. DBFNet [21] uses bilateral filtering to focus on enhancing the extraction of boundary information and filtering noise using a combination of feature nonlinearities at different scales. OANet [22] is a semantic segmentation network for marking directional attention, which uses asymmetric convolution to focus on features in different directions and explore their anisotropy, enhancing the semantic feature representation within the model. All the above methods fuse local features to form global feature information based on CNN, rather than directly encode global features.

### 2.2. Work on Transformer

In recent years, Transformer [23] has moved from natural language processing to image processing. Compared with CNN to obtain local feature information through convolution operation, Transformer can realize remote image dependence and encode global feature information by using a self-attention mechanism [24]. Transformer will divide the whole image into multiple tokens, and the multi-head attention mechanism is used to explore the feature relationship among all tokens and to encode the global feature. Its success in global relational feature modeling provides a new idea for many research fields. SETR [25] first applied the Transformer architecture to the field of image segmentation and introduced a sequence-to-sequence classification model, which greatly improved the problem of difficulty in obtaining global receptive fields. Segmenter [26] designs the encoder–decoder structure using Transformer and transforms the processed tokens into pixel-level annotations for encoding. PVT [27] combines Transformer with a pyramid structure, which can reduce the computation of the model by using the pyramid module while training the features intensively. SegFormer [28] was used for the design of a layered Transformer architecture with an improved MLP decoder to fuse the features of different layers to improve the classification performance of the network. RSSFormer [29] embeds Transformer into HRNet and designs adaptive multi-headed attention and MLP with dilation convolution to increase the foreground saliency of remote sensing images. DAformer [30] designs a multi-level context-aware structure based on Transformer, which effectively enhances the contextual semantic interaction. However, since the Transformer places each attention computation on the entire image, the rapidly increasing training cost will hinder the application of the model when the image size is large [31]. The Swin transformer [32] divides the image into different windows and restricts the attention computation to the windows, which makes it only linear in complexity. The shifting of window partitions between layers is a key component of the Swin Transformer architecture, where the shifted windows connect the windows of the previous layer and significantly improve the modeling capabilities. With only linear computational complexity, the Swin Transformer delivers advanced performance in a variety of fields such as video processing [33], image generation [34], and image segmentation [35].

## 3. Methods

In this section, we will explain in detail the architecture of TMNet and the main modules that make up this network architecture.

### 3.1. Network Structure

**Figure 1:**
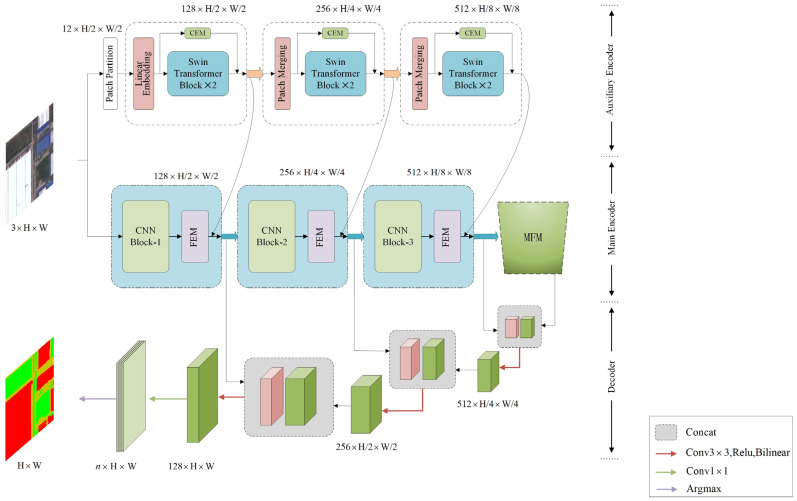
The overall structure of TMNet.

The overall network architecture of TMNet is shown in Figure 1. The dual encoder-decoder architecture is adopted, in which the CNN module with powerful feature extraction capability is used as the main encoder, and the Swin Transformer module with global feature information encoding capability is used as the auxiliary encoder. For a given remote sensing image, the image is first divided into different blocks and uses linear embedding to ensure the feature size is 128 × H/2 × W/2 and enhance the semantic features, and then the Swin Transformer module is used to enhance feature global modeling and feature extraction. In addition, we added CEM to the Swin transformer block to enhance the feature relationships between channels, reduce dimensionality by downsampling features in the patch merging layer, and increase dimensionality by splicing features. The three output sizes of the auxiliary encoder are 128 × H/2 × W/2, 256 × H/4 × W/4, and 512 × H/8 × W/8 and are summed with the corresponding output of the main encoder, enhancing the global modeling capability of the network. In the main encoder, the CNN module is used to extract the image backbone features, the FEM enhances the feature extraction capability of the main encoder by calculating the relationship between the input data and the updatable feature storage units, and the MFM fuses features at different scales through softpool of different sizes, especially enhancing the extraction capability of small-scale features. The decoder is primarily implemented by convolution and bilinear interpolation upsampling, and we use skip connections to increase the contextual relationship between the encoder and decoder. After decoding 3 times, the 1 × 1 convolutional layer and argmax function are used to obtain the final predicted image.

### 3.2. Main Encoder

#### 3.2.1. CNN Blocks

CNNs have been successful in many fields due to their powerful feature extraction capabilities. CNN Blocks have three modules, and Table 1 shows the detailed parameters of each module. The order of the parameters in Conv2d is in_channel, out_channels, kernel_size, step size, and padding, and all other parameters are default. maxPool2d(2) indicates that max pooling uses convolutional kernels to halve the input size, and BN indicates BatchNormal.

#### 3.2.2. Multi-Scale Feature Fusion Module

**Figure 2:**
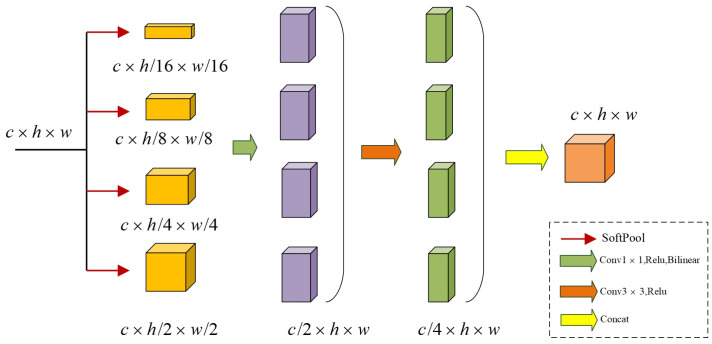
The structure of multi-scale feature fusion module.

CNN-based models perform convolutional operations for downsampling during feature extraction, which can easily lead to the loss of small-scale features. To solve this problem, we propose a multiscale feature fusion module (MFM), as shown in Figure 2. We ingeniously combine Softpool [36] with convolution operations, and Softpool obeys a certain probability distribution to retain finer feature cues compared to Avgpool and Maxpool. MFM uses SoftPool to extract multi-scale features by downsampling at scales of 2, 4, 8, and 16, respectively. Then 1 × 1 convolution is used to make the finer semantic feature information more refined, and bilinear interpolation is used for upsampling to augment the features. Subsequently, 3 × 3 convolution is used, and feature concatenation is performed to fuse multi-scale semantic features for feature class recovery.

Softpool is computed as follows:

(1) $$\underset{a}{˜}\;=\sum_{i\in R}\frac{e^{a_{i}}*a_{i}}{\sum_{j\in R}e^{a_{j}}},$$

The MFM execution process can be expressed as follows:

(2) $$Y_{n}=\mathrm{Softpool}(X),\;T_{n}=\mathrm{Bi}(\sigma (\mathrm{Conv}1\times 1(Y_{n}))),\;W_{n}=\sigma (\mathrm{Conv}3\times 3(T_{n})),$$

Bi(·) stands for bilinear interpolation upsampling and σ represents the Relu function. The input features $X\in R^{c\times h\times w}$, after the Softpool $Y_{n}\in R^{c\times h/n\times w/n}$, where *n* is the downsampling rate. The features $Y_{n}$ are convolved by the 1 × 1 convolution kernel and the Relu function, the number of channels is halved, and the size becomes the original size after upsampling bilinear interpolation, $T_{n}\in R^{c/2\times h\times w}$. For further feature extraction, after convolution and Relu activation function, the number of channels becomes one-fourth of the original number of channels. Finally, four different scale features are connected for multi-scale feature fusion and the number of channels becomes the output of the module, $W_{n}\in R^{c/4\times h\times w}$. Finally, four different scale features are concatenated for multi-scale feature fusion, and $Z\in R^{c\times h\times w}$ the number of channels resets to the original as the output result of this module.

(3) $$Z=[W_{16},W_{8},W_{4},W_{2}]$$

[·] Representing dimensional concatenated.

#### 3.2.3. Feature Enhancement Module

**Figure 3:**
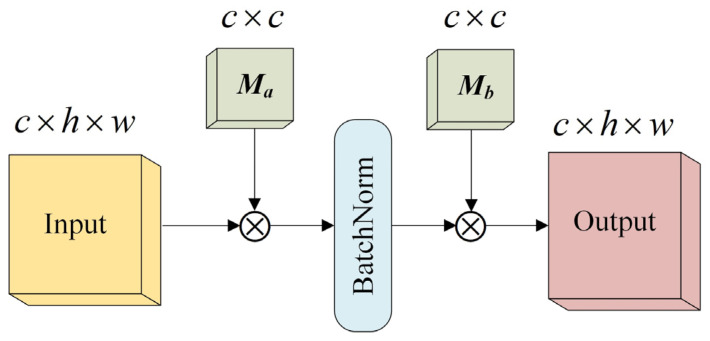
Feature enhancement module.

CNN has a strong feature detection capability, but the understanding of features is insufficient and the spatial resolution of features gradually decreases with the increase in depth and number of layers, which hinders the prediction capability of the target location in remote sensing images [37]. To address this problem, this paper proposes a feature enhancement module (FEM) to deepen the relationship between features and improve the performance of the encoder. The FEM is added after each CNN module to further learn the output features of each module, as shown in Figure 3. The FEM is designed with two updateable feature storage units to retain the feature relationships of the data, which can better learn the distribution weight relationships of the features with constant feature size, and adding normalization in the two parameter units can further densify the features and accelerate the convergence speed of the model. At the same time, the FEM parameters are small, which can better trade-off the efficiency and accuracy of the model. FEM can be expressed as follows:

(4) $$O=M_{b}⊗N(M_{a}⊗F),$$

where the input features are $F\in R^{c\times h\times w}$, and the output features are $O\in R^{c\times h\times w}$, learnable features storage units are $M_{a}\in R^{c\times c}$, $M_{b}\in R^{c\times c}$, N(⋅) denotes the BatchNorm, and ⊗ denotes the matrix multiplication.

### 3.3. Auxiliary Encoder

#### 3.3.1. Swin Transformer

**Figure 4:**
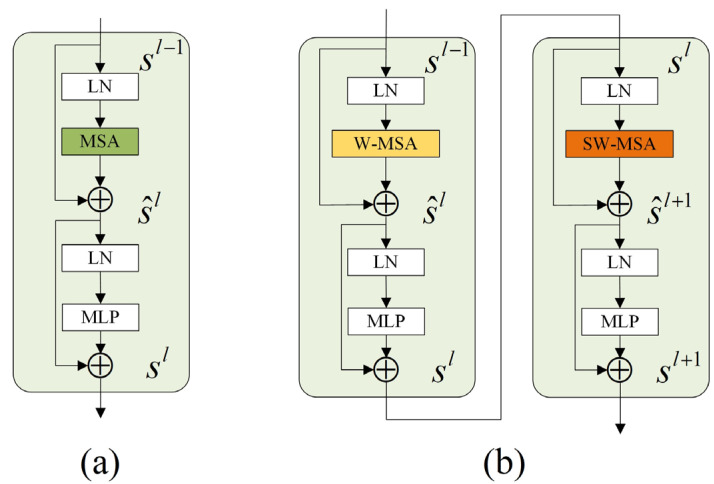
Transformer and Swin Transformer. (**a**) Standard Transformer, (**b**) Swin Transformer.

The standard transformer consists of a multiple attention mechanism (MSA), a multilayer perceptron (MLP), and layer normalization (LN), as shown in Figure 4a. MSA is the key mechanism in the Transformer that takes the input token and performs the feature relationship operation and encodes the positional relationship between different features in it to establish the global relationship. For the standard transformer, the output of l-layer sl can be expressed as:

(5) ^sl =MSA(LN(sl–1))+s,sl=MLP(LN(^sl))+^sl,

The transformer uses MSA to compute the global feature information among all tokens, resulting in a computational complexity that is linearly related to the image area size, which is not friendly to its practical application. Unlike the transformer module, the Swin Transformer applies the attention mechanism to the different windows divided from the image and enhances the global feature relationships by shifting between windows, as shown in Figure 4b, where the Swin Transformer consists of a window multihead self-attention (W-MSA) module connected to a shift window multihead self-attention (SW-MSA). In the Swin Transformer, the image is divided into different patches according to their positions by patch partition, and the input information is converted into sequences and embedded as tokens; then the sequences are encoded, the dimensions are changed by the linear embedding layer, and the uniformly encoded tokens are passed through the Swin Transformer block and the patch merging layer to generate a global feature relationship representation. The Swin transformer uses multihead self-attention to divide the features into multiple heads, where each head is responsible for exploring different local information relationships, and the global information is obtained by fusing the resultant letters from different heads. This can be expressed as follows:

(6) ^sl=W-MSA(LN(sl–1))+sl–1,sl=MLP(LN(^sl))+^sl,^sl+1=SW-MSA(LN(sl))+sl,sl+1=MLP(LN(^sl+1))+^sl+1,

where ^sl and $s^{l}$ denote the output of the lth (S)W-MSA and MLP modules, respectively. The number of layers corresponding to each stage of the Swin transformer configured in this experiment is (2,2,2), and the number of heads corresponding to each stage is (3,6,12), and the partition window size is set to 8.

#### 3.3.2. Channel Enhancement Module

**Figure 5:**
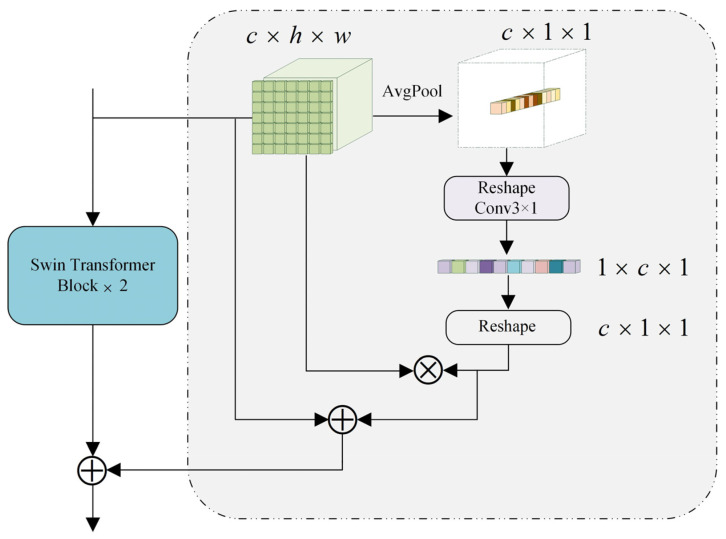
Channel enhancement module.

The Swin Transformer constructs patch token relationships in a limited number of windows, significantly reducing the computational complexity. However, this approach limits the window-to-window channel modeling capability to some extent, even with the strategy of the window and shift window cross execution. In this paper, we propose the channel enhancement module (CEM) to further enhance the window-to-window feature relationship interaction, compress the deep features in each channel through Avgpool, and use 3 × 1 convolution to interact with the channel features to extract more accurate spatial location information. The design of the CEM considers not only patch-to-patch but also channel-to-channel relationships, compensating for the window-to-window modeling capability limited by Swin Transformer and making the converter more suitable for image segmentation tasks. Unlike other methods [38,39], which divide windows by simply changing the Swin Transformer, our proposed CEM learns the deep relationships between channels to make its feature weight distribution more accurate. The CEM is shown in Figure 5. Channel information is first obtained by Avgpool, which is calculated as follows:

(7) vk=1wh∑i=0w−1∑j=0h−1^zk(i,j),

For the average pooled features, we convert the dimensions to 1 × *c* × 1 and then perform a convolution operation using a 3 × 1 convolution kernel and matrix multiply it with the original features to enhance their spatial characteristics. This is represented as follows:

(8) $$F=s^{l}⊗R(\mathrm{conv}3\times 1(R(v))⊗s^{l},$$

$s^{l}$ represents the output of the *l*th Swin Transformer block, R(⋅) represents the Reshape, and $v\in R^{c\times 1\times 1}$ represents the feature matrix after Avgpool.

### 3.4. Decoder

The decoder primarily decodes the features by convolutional operation and upsampling and skips the link with the main encoder in its process to enhance the global context information, as shown in the decoder part of Figure 1. Firstly, the MFM module output features are spliced with the CNN-Block3 module output to enhance the feature information interaction, followed by a convolutional kernel of 3 × 3, and after the Relu function for feature decoding and channel size reduction, the feature size is doubled by image up-sampling using linear interpolation. After three upsamplings, the feature size is changed to the original image size, and then the features are further refined using a 3 × 3 convolution kernel, and the number of feature channels is reduced to the number of image classes, and the prediction results are output by argmax function.

### 3.5. Loss Function

The loss function applied in this paper is a combination of Cross-entropy loss and Dice loss, where Cross-entropy loss primarily measures the difference in accuracy between pixels and Dice loss measures the accuracy of category regions.

Cross-entropy loss is expressed as follows:

(9) $$L_{\mathrm{ce}}=–\frac{1}{N}\sum_{i=0}^{N–1}\sum_{k=0}^{K–1}y_{i,k}lnp_{i,k},$$

where *N* is the number of samples and *K* represents the number of categories, *p_i,k_* represents the prediction probability of the *k*th category in the *i*th sample, and *y_i,k_* indicates whether the prediction result corresponds to the true label.

The Dice loss is expressed as follows:

(10) $$L_{dice}=1-\frac{2\sum_{i=0}^{N-1}\sum_{k=0}^{K-1}g_{i}^{k}s_{i}^{k}}{\sum_{i=0}^{N-1}\sum_{k=0}^{K-1}g_{i}^{k}+\sum_{i=0}^{N-1}\sum_{k=0}^{K-1}s_{i}^{k}},$$

*s_i_^k^*, *g_i_^k^* denote the predicted classification result and the actual label value of the image for the *k*th class of the *i*th sample, respectively.

The total loss function is expressed as:

(11) $$L_{\mathrm{total}}=L_{\mathrm{ce}}+L_{\mathrm{dice}},$$

## 4. Experiments and Results

To verify the efficiency of TMNet in the field of remote sensing image segmentation, we conducted experiments on two publicly available remote sensing image datasets and compared them with other classical methods.

### 4.1. Datasets

The two publicly available datasets used in this experiment are the Wuhan Dense Labeling Dataset (WHDLD) [40,41] and the Potsdam dataset [42].

The WHDLD was captured by the Gaofen-1 and ZY-3 satellites in Wuhan and consists of 4940 RGB images of size 256 × 256 that were fused and upsampled to a resolution of 2 m. There are six image categories in the WHDLD, including bare soil, buildings, pavement, vegetation, roads, and water, which have been classified pixel by pixel. The Potsdam dataset has 38 remote sensing images of size 6000 × 6000 with a resolution of 5 cm, covering 3.42 km^2^ of the building complex structures in Potsdam. The Potsdam dataset contains six categories: Impervious surface, building, low vegetation, tree, car, and clutter/background. In this paper, only 14 RGB images are selected for the experiment (image IDs: 2_13, 2_14, 3_13, 3_14, 4_13, 4_14, 4_15, 5_13, 5_14, 5_15, 6_14, 6_15, 7_13), and similar to WHDLD, to facilitate the experiment, we cropped the image size of the potsdam dataset to 256 × 256, for a total of 6877 images. We divided the training, validation, and test sets of these two datasets in the ratio of 6:2:2. To make the model more accurate, we used random rotation, flip, and Gaussian noise for data enhancement.

### 4.2. Training Setup and Evaluation Index

TMNet is built by PyTorch. We train the network using the SGD optimizer, where the momentum term is set to 0.9, the weights are decayed to 1 × 10^−4^, and for the learning rate, we set its initial value to 0.01 and halve the learning rate every 20 epochs to ensure the model reaches the optimal value faster. The experiments included are implemented on an NVIDIA GeForce RTX 3090 GPU with 24 GB RAM. The batch size is 16 and the maximum epoch is 150. We evaluate the performance of the model using mean intersection over union (MIOU), Mean F1 (MF1), and Mean Pixel Accuracy (MPA). The three evaluation metrics are calculated from the confusion matrix, which has four components: True positive (*TP*), false positive (*FP*), true negative (*TN*), and false negative (*FN*). Based on the above four terms, IOU is defined as the degree of overlap between the predicted and true values of an image and is calculated as follows:

(12) $$\mathrm{IOU}=\frac{\mathrm{TP}}{\mathrm{TP}+\mathrm{FP}+\mathrm{FN}},$$

The F1 score for each category is expressed as follows:

(13) $$F1=2\times \frac{precision\times recall}{precision+recall},$$

where *Precision* = *TP*/(*TP* + *FP*) and *Recall* = *TP*/(*TP* + *FN*). MIOU represents the mean of IOU for all categories, MF1 represents the mean of F1 for all categories, and MPA represents the mean value of precision for all categories, where we use MIOU as the main evaluation metric.

### 4.3. Comparison Results on WHDLD

**Figure 6:**
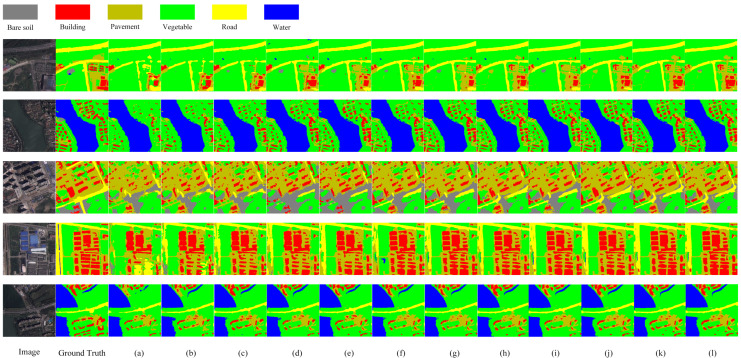
Results for all methods on WHDLD: (**a**) DFANet; (**b**) DenseASPP; (**c**) PSPNet; (**d**) DUNet; (**e**) MAUNet; (**f**) MUNet; (**g**) MSCFF; (**h**) Deeplabv3plus; (**i**) HRNet; (**j**) SegFormer; (**k**) HRVit; (**l**) TMNet.

In this section, we compare TMNet with other classical semantic segmentation networks, including DFANet [43], DenseASPP [15], PSPNet [16], Deeplabv3plus [44], DFANe-t [43], DUNet [45], MAUNet [46], MSCFF [47], MUNet [48], HRNet [20], SegFormer [28], and HRVit [49] where DenseASPP, PSPNet, and Deeplabv3plus use resnet101 as the backbone. The experimental results are shown in Table 2 and Figure 6.

From Table 2, it can be seen that the experimental results of TMNet outperform the other networks, where DFANet is based on pure CNN architecture, DenseASPP improves its global modeling capability by using convolution with different scaling rates, PSPNet utilizes context through pyramid pooling module, and all the above methods use local features to aggregate contextual information. The MSCFF uses trainable convolutional filters to densify the feature map and enhance small-scale features, MAUNet subdivides features at different scales by increasing the number of downsampling and attention mechanisms, which cannot achieve multi-scale feature fusion compared with TMNet, and Deeplabv3plus uses atrous space pyramidal pooling and applies deep separable convolution, but the performance is still inferior to TMNet. SegFormer designs a hierarchical Transformer architecture and fuses features at different scales using an improved MLP decoder. HRVit uses Transformer to design cascading converters that generate multi-scale feature representations. However, the lack of contextual information interaction makes its performance inferior to that of TMNet. In addition, we compared the parameters and FLOPs of each method, and TM performed moderately well in both aspects, indicating that TMNet does not simply pile up computational effort to obtain high accuracy. Compared with the three advanced models of HRNet, SegFormer, and HRVit, TMNet improves MIOU by 0.80%, 0.78%, and 0.52% with only 41.77%, 43.41%, and 71.80% of HRNet, SegFormer, and HRVit in terms of parameters. Compared to FLOPs, TMNet is 3.65% and 39.95% less than HRNet and SegFormer. Figure 6 shows the prediction results of each method on the WHDLD. From Figure 6, it can be seen that the prediction results of TMNet are closest to the real image and better in terms of small-scale features and edge details. The first row of Figure 6 shows that TMNet achieves the best classification results in the road category due to the powerful global modeling capability of the Swin Transformer that enhances the feature extraction ability of the network. For the more difficult-to-classify bare soil category, TMNet also has good classification results for it, as can be seen in the third row of Figure 6. This shows the excellent performance of TMNet in the semantic segmentation of remote sensing images.

### 4.4. Comparison Results on Potsdam Dataset

**Figure 7:**
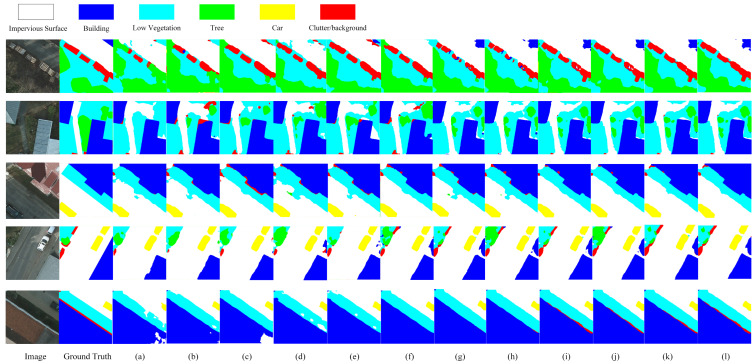
Results for all methods on Potsdam dataset: (**a**) DFANet; (**b**) MAUNet; (**c**) DenseASPP; (**d**) PSPNet; (**e**) DUNet; (**f**) Deeplabv3plus; (**g**) HRNet; (**h**) MSCFF; (**i**) MUNet; (**j**) HRVit; (**k**) SegFormer; (**l**) TMNet.

Table 3 shows the segmentation results of each method on the Potsdam dataset, which further proves the effectiveness of TMNet in the semantic segmentation study of remote sensing images as MIOU, MF1, and MPA reach 68.15%, 79.91%, and 78.77%, respectively, which are higher than other methods. Due to the difference in dataset resolution, the segmentation accuracy of Potsdam is better than that of WHDLD. Figure 7 shows the experimental prediction results of each method on the Potsdam dataset, and we can see that the classification results of TMNet are better than other methods, for example, it is obvious from the first and fifth rows that TMNet is significantly better than other methods in the prediction of clutter/background category, which demonstrates the performance of TMNet.

### 4.5. Ablation Study

In this section, to verify the effectiveness of each module proposed in this paper, we conducted ablation experiments on WHDLD with the CNN blocks and decoder as the baseline, and the results are shown in Table 4. SW stands for Swin Transformer.

#### 4.5.1. Effect of SW and MFM

**Figure 8:**
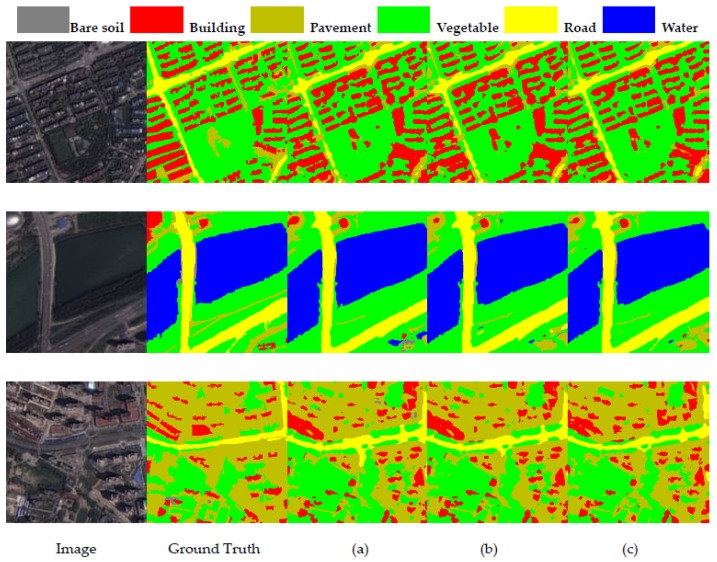
Results of ablation experiments: (**a**) Baseline; (**b**) Baseline + MFM; (**c**) Baseline + MFM + SW.

Table 4’s first three rows demonstrate that the addition of MFM improves MIOU by 1.18%, which is primarily due to the fact that MFM combines low-level spatial features of images at different scales into high-level semantic features, which enhances multi-scale feature information and increases the network’s ability to detect minute features. The MIOU improves by 1.03% when SW is added, which is primarily due to the powerful global modeling capability of SW and the enhanced global context acquisition capability of the network using the dual branch structure. Figure 8 shows the results, from which it can be seen that after adding MFM and SW again, the segmentation effect capability is significantly improved, especially for small, detailed features of the image. For example, the accuracy improvement of the Road category detection results in the first and second rows of Figure 8 is more obvious.

#### 4.5.2. Effect of FEM and CEM

**Figure 9:**
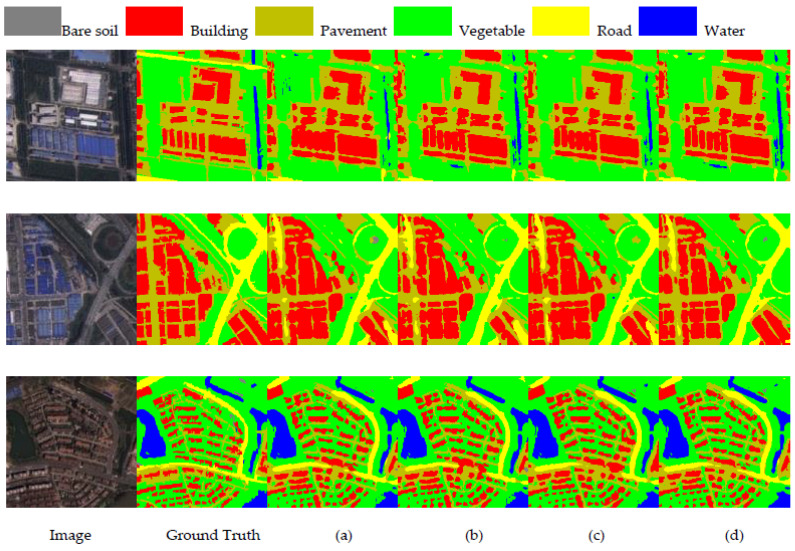
Results of ablation experiments: (**a**) Baseline_1; (**b**) Baseline_1 + FEM; (**c**) Baline_1 + CEM; (**d**) Baseline_1 + CEM + FEM.

We refer to baseline + GD + SW as baseline_1. As shown in the last four rows of Table 4, the MIOU improves by 0.37% when FEM is added, which is due to the fact that FEM combines the input data with learnable parameters to further enhance the feature extraction capability. When CEM is added, the MIOU is improved by 0.48% due to the enhanced window-to-window modeling capability of CEM by focusing on the relationship between channels. When both modules are added, MIOU is improved by 0.82%, which shows the effect of FEM and CEM on network performance enhancement. Figure 9 shows the segmentation results. From the first row, we can see that the building category is more clearly segmented after adding the two modules, and the confusion between the Road and Pavement categories in the second row has been greatly improved.

## 5. Conclusions

In this paper, a two-branch multi-scale semantic segmentation network named TMNet for remote sensing images is proposed. It adopts the encoder–decoder structure, enhances the global contextual interaction of the network with the dual-branch structure of the Swin Transformer to make up for the lack of global modeling capability of CNN, and the MFM is proposed to improve the fusion of features at different scales. In addition, FEM and CEM aim to enhance the feature capture capability of the network through feature information relationship calculation. However, the TMNet prediction contours still cannot be fully fitted with the actual results. We will explore new methods to improve, and also work on network simplification to improve computational efficiency.

## Figures and Tables

**Table 1:** CNN block parameters.

Name	Input Size	Layer Structure	Out Size
CNN block-0	3 × H × W	Conv2d(3,64,3,2,1), Relu	128 × H/2 × W/2
		Conv2d(64,96,3,1,1), BN, Relu	
		Conv2d(96,128,3,1,1), BN, Relu	
CNN block-1	128 × H/2 × W/2	MaxPool2d(2)	256 × H/4 × W/4
		Conv2d(128,192,3,1,1), BN, Relu	
		Conv2d(192,256,3,1,1), BN, Relu	
CNN block-2	256 × H/4 × W/4	MaxPool2d(2)	512 × H/8 × W/8
		Conv2d(256,384,3,1,1), BN, Relu	
		Conv2d(384,512,3,1,1), BN, Relu	

**Table 2:** Results of comparison with other methods on WHDLD (%).

Method	Parameters (M)	FLOPs (G)	MIOU	MF1	MPA	IOU			
						Bare Soil	Building	Vegetable	Water
DFANet	2.32	0.947	54.93	68.46	67.88	49.46	47.75	77.02	90.56
DenseASPP	46.04	48.178	55.94	69.23	67.60	49.04	47.14	78.63	92.41
PSPNet	72.31	70.058	56.41	69.88	68.44	48.18	53.43	76.28	91.74
DUNet	29.21	30.889	56.86	70.27	68.44	49.27	54.43	76.42	91.97
MAUNet	14.55	41.263	57.12	70.41	69.56	50.88	49.79	78.99	92.70
MUNet	7.77	13.756	58.50	71.65	70.20	51.40	54.45	79.38	93.17
MSCFF	51.95	140.680	59.87	72.88	70.82	53.60	56.86	79.41	94.05
Deeplabv3plus	59.34	22.243	60.37	73.32	71.61	56.54	53.04	80.80	93.96
HRNet	49.20	49.302	60.43	73.21	71.51	55.66	54.01	80.96	93.97
SegFormer	47.34	79.105	60.45	73.33	71.55	55.26	52.91	81.16	94.18
HRVit	28.62	27.415	60.71	73.57	71.82	55.17	53.42	81.12	93.89
TMNet	20.55	47.499	61.23	74.14	72.57	55.77	54.06	81.19	94.28

**Table 3:** Results of comparison with other methods on Potsdam dataset (%).

Method	MIOU	MF1	MPA	IOU					
				Impervious Surface	Building	Low Vegetation	Tree	Car	Clutter/Background
DFANet	61.05	73.70	72.30	70.92	77.79	60.18	63.49	73.16	20.75
MAUNet	62.31	75.01	73.15	70.55	77.75	61.30	65.53	74.07	24.66
DenseASPP	62.93	75.92	75.24	75.00	80.02	61.29	56.69	72.53	32.02
PSPNet	63.76	76.37	75.51	74.28	82.83	62.01	59.49	73.99	29.99
DUNet	64.70	77.00	75.65	76.70	83.17	63.09	58.47	76.29	30.45
Deeplabv3plus	65.32	77.49	77.46	75.73	83.90	63.06	63.46	75.51	30.25
HRNet	66.22	78.18	77.49	76.62	83.51	64.66	61.53	77.28	33.69
MSCFF	66.35	78.36	78.88	75.28	84.31	64.95	63.21	77.83	32.48
MUNet	66.60	78.42	77.05	76.26	83.10	64.13	62.94	77.44	32.31
HRVit	67.33	79.72	77.70	76.70	84.86	64.71	64.05	77.65	35.99
SegFormer	67.43	79.07	78.43	76.61	84.66	65.08	63.70	78.17	36.36
TMNet	68.15	79.91	78.77	78.63	84.20	66.65	63.74	78.57	37.14

**Table 4:** Results of ablation experiments on WHDLD (%).

Method	MIOU	MF1	MPA
Baseline	58.20	71.43	69.95
Baseline + MFM	59.38	72.36	71.17
Baseline + MFM + SW	60.41	73.42	72.08
Baseline + MFM + SW + FEM	60.78	73.71	72.59
Baseline + MFM + SW + CEM	60.89	73.82	72.34
Baseline + MFM + SW + FEM + CEM	61.23	74.14	72.57

## Data Availability

The WHDLD can be obtained from https://sites.google.com/view/zhouwx/dataset. The Potsdam dataset can be obtained from https://www.isprs.org/education/benchmarks/UrbanSemLab/Default.aspx.

## References

[1] Morin E., Herrault P.-A., Guinard Y., Grandjean F., Bech N. (2022). The promising combination of a remote sensing approach and landscape connectivity modelling at a fine scale in urban planning. Ecol. Indic..

[2] Qing Y., Ming D., Wen Q., Weng Q., Xu L., Chen Y., Zhang Y., Zeng B. (2022). Operational earthquake-induced building damage assessment using CNN-based direct remote sensing change detection on superpixel level. Int. J. Appl. Earth Obs. Geoinf..

[3] Lu W., Zhao L., Xu R. (2023). Remote sensing image processing technology based on mobile augmented reality technology in surveying and mapping engineering. Soft Comput..

[4] Wang P., Bayram B., Sertel E. (2022). A comprehensive review on deep learning based remote sensing image super-resolution methods. Earth-Sci. Rev..

[5] De Geus D., Dubbelman G. Intra-Batch Supervision for Panoptic Segmentation on High-Resolution Images. Proceedings of the IEEE/CVF Winter Conference on Applications of Computer Vision.

[6] Zhao Z., Long S., Pi J., Wang J., Zhou L. Instance-specific and Model-adaptive Supervision for Semi-supervised Semantic Segmentation. Proceedings of the IEEE/CVF Conference on Computer Vision and Pattern Recognition.

[7] Long J., Shelhamer E., Darrell T. Fully convolutional networks for semantic segmentation. Proceedings of the IEEE Conference on Computer Vision and Pattern Recognition.

[8] Ronneberger O., Fischer P., Brox T. U-net: Convolutional networks for biomedical image segmentation. Proceedings of the Medical Image Computing and Computer-Assisted Intervention–MICCAI 2015: 18th International Conference.

[9] Badrinarayanan V., Kendall A., Cipolla R. (2017). Segnet: A deep convolutional encoder-decoder architecture for image segmentation. IEEE Trans. Pattern Anal. Mach. Intell..

[10] Chen X., Li Z., Jiang J., Han Z., Deng S., Li Z., Fang T., Huo H., Li Q., Liu M. (2020). Adaptive effective receptive field convolution for semantic segmentation of VHR remote sensing images. IEEE Trans. Geosci. Remote Sens..

[11] Zhao Y., Zhang X., Feng W., Xu J. (2022). Deep Learning Classification by ResNet-18 Based on the Real Spectral Dataset from Multispectral Remote Sensing Images. Remote Sens..

[12] Chen J., Lu Y., Yu Q., Luo X., Adeli E., Wang Y., Lu L., Yuille A.L., Zhou Y. (2021). Transunet: Transformers make strong encoders for medical image segmentation. arXiv.

[13] Ding L., Tang H., Bruzzone L. (2020). LANet: Local attention embedding to improve the semantic segmentation of remote sensing images. IEEE Trans. Geosci. Remote Sens..

[14] Chen L.-C., Papandreou G., Kokkinos I., Murphy K., Yuille A.L. (2017). Deeplab: Semantic image segmentation with deep convolutional nets, atrous convolution, and fully connected crfs. IEEE Trans. Pattern Anal. Mach. Intell..

[15] Yang M., Yu K., Zhang C., Li Z., Yang K. Denseaspp for semantic segmentation in street scenes. Proceedings of the IEEE Conference on Computer Vision and Pattern Recognition.

[16] Zhao H., Shi J., Qi X., Wang X., Jia J. Pyramid scene parsing network. Proceedings of the IEEE Conference on Computer Vision and Pattern Recognition.

[17] Dong R., Pan X., Li F. (2019). DenseU-net-based semantic segmentation of small objects in urban remote sensing images. IEEE Access.

[18] Yu C., Wang J., Peng C., Gao C., Yu G., Sang N. Bisenet: Bilateral segmentation network for real-time semantic segmentation. Proceedings of the European Conference on Computer Vision (ECCV).

[19] Hou J., Guo Z., Wu Y., Diao W., Xu T. (2022). BSNet: Dynamic Hybrid Gradient Convolution Based Boundary-Sensitive Network for Remote Sensing Image Segmentation. IEEE Trans. Geosci. Remote Sens..

[20] Wang J., Sun K., Cheng T., Jiang B., Deng C., Zhao Y., Liu D., Mu Y., Tan M., Wang X. (2020). Deep high-resolution representation learning for visual recognition. IEEE Trans. Pattern Anal. Mach. Intell..

[21] Wu L., Fang L., Yue J., Zhang B., Ghamisi P., He M. (2022). Deep Bilateral Filtering Network for Point-Supervised Semantic Segmentation in Remote Sensing Images. IEEE Trans. Image Process.

[22] Wang J., Feng Z., Jiang Y., Yang S., Meng H. (2023). Orientation Attention Network for semantic segmentation of remote sensing images. Knowl.-Based Syst..

[23] Dosovitskiy A., Beyer L., Kolesnikov A., Weissenborn D., Zhai X., Unterthiner T., Dehghani M., Minderer M., Heigold G., Gelly S. (2020). An image is worth 16×16 words: Transformers for image recognition at scale. arXiv.

[24] Vaswani A., Shazeer N., Parmar N., Uszkoreit J., Jones L., Gomez A.N., Kaiser Ł., Polosukhin I. (2017). Attention is all you need. Adv. Neural Inf. Process. Syst..

[25] Zheng S., Lu J., Zhao H., Zhu X., Luo Z., Wang Y., Fu Y., Feng J., Xiang T., Torr P.H. Rethinking semantic segmentation from a sequence-to-sequence perspective with transformers. Proceedings of the IEEE/CVF Conference on Computer Vision and Pattern Recognition.

[26] Strudel R., Garcia R., Laptev I., Schmid C. Segmenter: Transformer for semantic segmentation. Proceedings of the IEEE/CVF International Conference on Computer Vision.

[27] Wang W., Xie E., Li X., Fan D.-P., Song K., Liang D., Lu T., Luo P., Shao L. Pyramid vision transformer: A versatile backbone for dense prediction without convolutions. Proceedings of the IEEE/CVF International Conference on Computer Vision.

[28] Xie E., Wang W., Yu Z., Anandkumar A., Alvarez J.M., Luo P. (2021). SegFormer: Simple and efficient design for semantic segmentation with transformers. Adv. Neural Inf. Process. Syst..

[29] Xu R., Wang C., Zhang J., Xu S., Meng W., Zhang X. (2023). Rssformer: Foreground saliency enhancement for remote sensing land-cover segmentation. IEEE Trans. Image Process..

[30] Hoyer L., Dai D., Van Gool L. Daformer: Improving network architectures and training strategies for domain-adaptive semantic segmentation. Proceedings of the IEEE/CVF Conference on Computer Vision and Pattern Recognition.

[31] Ren H., Dai H., Dai Z., Yang M., Leskovec J., Schuurmans D., Dai B. (2021). Combiner: Full attention transformer with sparse computation cost. Adv. Neural Inf. Process. Syst..

[32] Liu Z., Lin Y., Cao Y., Hu H., Wei Y., Zhang Z., Lin S., Guo B. Swin transformer: Hierarchical vision transformer using shifted windows. Proceedings of the IEEE/CVF International Conference on Computer Vision.

[33] Liu Z., Ning J., Cao Y., Wei Y., Zhang Z., Lin S., Hu H. Video swin transformer. Proceedings of the IEEE/CVF Conference on Computer Vision and Pattern Recognition.

[34] Lin A., Chen B., Xu J., Zhang Z., Lu G., Zhang D. (2022). Ds-transunet: Dual swin transformer u-net for medical image segmentation. IEEE Trans. Instrum. Meas..

[35] Wang W., Chen W., Qiu Q., Chen L., Wu B., Lin B., He X., Liu W. (2023). CrossFormer++: A Versatile Vision Transformer Hinging on Cross-scale Attention. arXiv.

[36] Stergiou A., Poppe R., Kalliatakis G. Refining activation downsampling with SoftPool. Proceedings of the IEEE/CVF International Conference on Computer Vision.

[37] Ye X., Xiong F., Lu J., Zhao H., Zhou J. M 2-Net: A Multi-scale Multi-level Feature Enhanced Network for Object Detection in Optical Remote Sensing Images. Proceedings of the 2020 Digital Image Computing: Techniques and Applications (DICTA).

[38] Vaswani A., Ramachandran P., Srinivas A., Parmar N., Hechtman B., Shlens J. Scaling local self-attention for parameter efficient visual backbones. Proceedings of the IEEE/CVF Conference on Computer Vision and Pattern Recognition.

[39] Dong X., Bao J., Chen D., Zhang W., Yu N., Yuan L., Chen D., Guo B. Cswin transformer: A general vision transformer backbone with cross-shaped windows. Proceedings of the IEEE/CVF Conference on Computer Vision and Pattern Recognition.

[40] Shao Z., Yang K., Zhou W. (2018). Performance evaluation of single-label and multi-label remote sensing image retrieval using a dense labeling dataset. Remote Sens..

[41] Shao Z., Zhou W., Deng X., Zhang M., Cheng Q. (2020). Multilabel remote sensing image retrieval based on fully convolutional network. IEEE J. Sel. Top. Appl. Earth Obs. Remote Sens..

[42] (2018). ISPRS Vaihingen 2D Semantic Labeling Dataset.

[43] Li H., Xiong P., Fan H., Sun J. Dfanet: Deep feature aggregation for real-time semantic segmentation. Proceedings of the IEEE/CVF Conference on Computer Vision and Pattern Recognition.

[44] Chen L.-C., Zhu Y., Papandreou G., Schroff F., Adam H. Encoder-decoder with atrous separable convolution for semantic image segmentation. Proceedings of the European Conference on Computer Vision (ECCV).

[45] Tian Z., He T., Shen C., Yan Y. Decoders matter for semantic segmentation: Data-dependent decoding enables flexible feature aggregation. Proceedings of the IEEE/CVF Conference on Computer Vision and Pattern Recognition.

[46] Sun Y., Bi F., Gao Y., Chen L., Feng S. (2022). A multi-attention UNet for semantic segmentation in remote sensing images. Symmetry.

[47] Li Z., Shen H., Cheng Q., Liu Y., You S., He Z. (2019). Deep learning based cloud detection for medium and high resolution remote sensing images of different sensors. ISPRS J. Photogramm. Remote Sens..

[48] Wieland M., Li Y., Martinis S. (2019). Multi-sensor cloud and cloud shadow segmentation with a convolutional neural network. Remote Sens. Environ..

[49] Gu J., Kwon H., Wang D., Ye W., Li M., Chen Y.-H., Lai L., Chandra V., Pan D.Z. Multi-scale high-resolution vision transformer for semantic segmentation. Proceedings of the IEEE/CVF Conference on Computer Vision and Pattern Recognition.

